# Optimization and Evaluation of Poly(lactide-*co*-glycolide) Nanoparticles for Enhanced Cellular Uptake and Efficacy of Paclitaxel in the Treatment of Head and Neck Cancer

**DOI:** 10.3390/pharmaceutics12090828

**Published:** 2020-08-30

**Authors:** Mohamed Haider, Amr Elsherbeny, Jayalakshmi Jagal, Anna Hubatová-Vacková, Iman Saad Ahmed

**Affiliations:** 1 Department of Pharmaceutics and Pharmaceutical Technology, College of Pharmacy, University of Sharjah, Sharjah 27272, UAE; u15100239@sharjah.ac.ae (A.E.); iahmed@sharjah.ac.ae (I.S.A.); 2 Research Institute of Medical & Health Sciences, University of Sharjah, Sharjah 27272, UAE; jayajagal@gmail.com; 3 Department of Pharmaceutics and Industrial Pharmacy, Faculty of Pharmacy, Cairo University, Cairo 11562, Egypt; 4 Department of Chemical Engineering, University of Chemistry and Technology Prague, Technická 5, Prague 6, 16628 Prague, Czech Republic; hubatova@vscht.cz

**Keywords:** paclitaxel, poly(lactide-*co*-glycolide), nanoparticles, quality-by-design, head and neck cancer

## Abstract

The particle size (PS) and encapsulation efficiency (EE%) of drug-loaded nanoparticles (NPs) may inhibit their cellular uptake and lead to possible leakage of the drug into the systemic circulation at the tumor site. In this work, ultra-high paclitaxel-loaded poly(lactide-*co*-glycolide) NPs (PTX-PLGA-NPs) with ultra-small sizes were prepared and optimized by adopting the principles of quality by design (QbD) approach. The optimized PTX-PLGA-NPs showed ultra-small spherical particles of about 53 nm with EE% exceeding 90%, a relatively low polydispersity index (PDI) of 0.221, an effective surface charge of −10.1 mV, and a 10-fold increase in the in vitro drug release over 72 h relative to free drug. The cellular viability of pharynx carcinoma cells decreased by almost 50% in 24 h following treatment with optimized PTX-PLGA-NPs, compared to only 20% from the free drug. The intracellular uptake of PTX-PLGA-NPs was highly favored, and the antitumor activity of PTX was remarkably improved with a reduction in its half maximal inhibitory concentration (IC_50_), by almost 50% relative to free drug solution. These results suggest that the optimal critical formulation parameters, guided by QbD principles, could produce PLGA-NPs with remarkably high EE% and ultra-small PS, resulting in enhanced cellular uptake and efficacy of PTX.

## 1. Introduction

Head and neck squamous cell carcinomas (HNSCC) are considered among the most common malignancies, and a leading cause of cancer death worldwide [1]. Although the etiological risk factors are well-documented and advances in diagnosis and therapy using different modalities have been made, the morbidity of HNSCC has not improved significantly over the past few decades [2,3]. Current treatment protocols for HNSCC involve surgical and radiation therapies, which are widely associated with disfigurement and severe toxicity, such as mucositis, dysphagia, xerostomia, radiation dermatitis, hematologic toxicity, neurotoxicity, and ototoxicity [4]. Several chemotherapeutic agents are used in the management of HNSCC, including platinum compounds, 5-fluorouracil and taxanes; however, these have moderate efficacy, and are usually combined with checkpoint inhibitors such as pembrolizumab [5].

Paclitaxel (PTX) is a diterpenoid pseudoalkaloid anticancer drug extracted from the Pacific Yew tree *Taxus brevifolia* [6]. Its chemotherapeutic effect is associated with its ability to bind to tubulin promoting microtubule assembly, stabilizing the existing microtubules and enhancing the action of tubulin dimers, therefore inhibiting their disassembly. This interferes with the late G2 mitotic phase of the cell cycle, and inhibits cellular replication suppressing the metastasis of cancer cells [7,8]. In addition, PTX may stimulate tumor necrosis factors, inhibit tumor-angiogenesis and induce cytokines and tumor-suppressor genes [8,9]. PTX has shown remarkable antitumor properties, especially in the treatment of lung, ovarian and breast cancers, with moderate efficacy on HNSCC [7,10,11,12]. Its poor water solubility, inadequate selectivity and significant systemic side effect have limited its intravenous (IV) administration for the treatment of HNSCC [13]. The currently approved PTX formulation Taxol^®^ (Bristol-Myers Squibb Company, Princeton, NJ, USA) is a solution of the drug in polyoxyethylated castor oil and ethanol (Cr/Et) administered as IV infusion, over three hours every three weeks. It has been associated with serious hypersensitivity reactions, catheter-related infections, neutropenia, extravasation and prolonged peripheral neuropathy [12]. Alternatively, Abraxane^®^ (Abraxis BioScience, LLC, Los Angeles, CA, USA) is a solvent-free albumin-bound PTX nanoparticles (NPs) suspension, with lower risks of hypersensitivity reactions and shorter period of administration; however, it is limited by elevated neurotoxicity side effects [14].

Intratumoral (IT) chemotherapy involves the injection of the anticancer agent directly into the solid tumor. This approach may be suitable for the treatment of HNSCC, as it increases the drug concentration at the tumor site and reduces its systemic toxicity [15,16]. Recently, IT injection using biodegradable thermosensitive hydrogels, in situ forming implants, liposomes and NPs has gained significant attention as an alternative to IV administration of chemotherapeutic agents, as they can provide localized controlled drug delivery [15,17,18,19]. It can also be used to expose large solid tumors to high concentrations of the drug prior to surgery [17].

The use of polymeric NPs as carriers for lipophilic drugs, including PTX, has significantly increased the therapeutic effect of those drugs, by improving their aqueous solubility, altering their biodistribution profile, and protecting them against enzymatic degradation [7,20]. NPs smaller than 100 nm can also passively target the tumor via enhanced permeability retention (EPR) effect, where appropriately sized particles extravasate through the tumor vasculature and accumulate locally, which sustains the drug concentration compared to the parent small molecule [16,21]. Moreover, given their miniature size, NPs possess a large surface area-to-volume ratio, resulting in considerable interaction with tumor cells, thus enhancing their cellular uptake and cytotoxic effect on those cells [22].

PLGA is an FDA-approved biodegradable and biocompatible copolymer, widely used in the fabrication of NPs, that can encapsulate and enhance the pharmaceutical characteristics of hydrophobic chemotherapeutic agents such as PTX [23], curcumin (CUR) [24] and tamoxifen [25]. The attractive features of PLGA-based NPs, such as small size, high structural integrity, colloidal stability, ease of fabrication, controlled release capability, and surface functionalization, make them very promising therapeutic delivery vehicles [25,26]. PLGA has a variety of types in terms of Mw and lactic acid to glycolic acid ratio [27]. Using PLGA with different Mw affects the release rate of the therapeutic agent, as it influences PS, EE% and polymeric degradation [28,29]. Moreover, changing the ratio of lactic to glycolic acid moieties in the copolymer affects its hydrophilicity as well as its biodegradation [8,30].

By controlling the degree of hydrophobicity and the Mw of the polymer along with other formulation parameters, the physico-chemical characteristics of PLGA-NPs can be efficiently tuned to fit the desired application according to its collective properties [8,31]. This can be readily achieved by formulation optimization techniques to determine the levels of variables from which a robust product with high quality characteristics may be produced.

The use of design of experiments (DoE) and QbD approaches enable a rational investigation of the influence of critical formulation/process variables on the relevant responses, by reducing the number of experimental runs, and identifying significant interactions between the independent variables, thus speeding up product development and reducing costs [32,33].

The aim of the present study is to fabricate and optimize ultra-high paclitaxel-loaded poly(lactide-*co*-glycolide) nanoparticles (PTX-PLGA-NPs) with ultra-small size, using the principles of QbD, to enhance the cellular uptake of PTX, and to form an extended-release drug depot in the form of PTX-PLGA-NPs at the tumor site, as a novel approach to increase the efficacy of PTX in the treatment of HNSCC, when injected in the form of an intratumoral injection (IT). PTX-PLGA-NPs were prepared using a modified nanoprecipitation technique and the influence of several formulation variables on the PS, PDI, zeta potential (ZP) and EE% were studied and optimized using D-optimal response surface design. The possible chemical interaction between the components used in the fabrication of the NPs was studied and the in vitro drug release from the optimal formulation was determined. The cytotoxic potential of PTX from the optimal NPs formulation on head and neck cancer cell lines was determined and the cellular uptake of the NPs was investigated by fluorescent microscopy, using CUR as a model hydrophobic fluorescent agent.

## 2. Materials and Methods

### 2.1. Materials

Paclitaxel (PTX), Mw = 853.906 g/mol, was obtained from LC laboratories (Woburn, MA, USA). Poly(d,l-lactide-*co*-glycolide) (PLGA) 50:50, Mw = 7000–17,000 Da (PLGA-7K), 24,000–38,000 Da (PLGA-24K) and 38,000–54,000 Da (PLGA-38K); Kolliphor^®^ P188 (Kol) (poly(ethylene glycol)-block-poly(propylene glycol)-block-poly(ethylene glycol); *N*-methyl pyrrolidone (NMP), acetonitrile HPLC grade 99% purity, Eagle’s minimum essential medium (MEM), fetal bovine serum (FBS) and curcumin (CUR, Mw = 368.38 g/mol), were purchased from Sigma-Aldrich Co. (St. Louis, MI, USA).

### 2.2. QbD Approach for Optimization of PTX-PLGA-NPs

The experimental design used in the optimization of PTX-PLGA-NPs was set up using Design-Expert^®^ software (Version 12.0, Stat-Ease Inc., Minneapolis, MN, USA). Two numerical and one categorical factors, amount (mg) of PLGA (*X*_1_), Kol % (*w*/*v*) (*X*_2_) and Mw of PLGA (*X*_3_) respectively, have been set as the critical material attributes (CMAs) for the experimental design, to investigate the effect of these formulation parameters on the selected CQAs. The levels of the independent variables were chosen to provide a maximal design space, and at the same time enable the feasible processing of the NPs (Table 1). The D-optimal response surface design was found to be appropriate to plan the levels of the independent variables and evaluate the output data with fewer number of experimental runs. On the other hand, four responses have been adopted to be tracked for the optimization of the studied factors: (1) PS (*Y*_1_), (2) PDI (*Y*_2_), (3) ZP (*Y*_3_), and (4) EE% (*Y*_4_) (Table 1). PTX-PLGA-NPs formulation was optimized for the responses *Y*_1_–*Y*_4_ with the target responses set at the smallest PS (<60 nm) and PDI and the highest ZP and EE%, to yield the system with the highest overall desirability value. The optimal independent variables were then used to prepare the optimal PTX-PLGA-NPs formulation.

The statistical design software yielded 22 experimental runs (formulations), as presented in Table 2. All experimental runs were conducted in random order to eliminate biased variance and increase the predictability of the model. All measurements were performed in triplicate (*n* = 3). All responses were simultaneously fitted to linear, two-factor interaction (2FI), and quadratic response surface models, and the resulting polynomial equations were statistically validated by the analysis of variance (ANOVA). Statistical parameters such as *p*-value, adjusted multiple correlation coefficient (*R*^2^-adj) and multiple correlation coefficient (*R*^2^) were determined to ensure the significance of the model. Moreover, 3D response surface plots were generated by Design-Expert^®^ software, to analyze the results graphically and determine the degree of interactions between the different factors for each response. The desirability function technique was used to optimize the formulation using numerical and graphical analysis. A desirability value of 0 was considered not acceptable, while a value closer to 1 corresponded to the desired response [34].

### 2.3. Preparation of PTX-PLGA-NPs

PTX-PLGA-NPs were prepared, as per the experimental design, using a modified nanoprecipitation technique [35]. Briefly, accurately weighed amounts (10–50 mg) of PLGA-7K, PLGA-24K or PLGA-38K were dissolved in 1 mL NMP, followed by adding 1 mg of PTX and the resulting solution was vortexed for 1 min, to ensure the complete dissolution of the drug and polymer. The organic solution was then added dropwise at a rate of 1 drop/min to 9 mL of an aqueous Kol solution (0.5–1% *w*/*v*), used as stabilizer, and the resulting mixture was kept under stirring at a rate of 750 rpm for 20 min. The formed NPs were centrifuged at 15,000 rpm for 20 min at 4 °C, using a high-speed centrifuge (Universal 320R Benchtop Centrifuge, Hettich, Beverly, MA, USA), and the separated NPs were washed with 1 mL of distilled water, to remove traces of excess Kol. The optimal NPs formulation obtained from the experimental design was also frozen at −80 °C for 4 h and lyophilized at −50 °C and 7 × 10^−2^ mbar for 48 h, using a freeze-dryer (Vir Tis Bench Top Pro, SP Scientific, Warminster, PA, USA) to yield re-dispersible dry powders. The vials containing the lyophilized PTX-PLGA-NPs were sealed immediately after removal from the freeze-dryer, wrapped in aluminum foil and stored at 4 °C in a refrigerator for further studies.

### 2.4. Physico-Chemical Characterization of PTX-PLGA-NPs

#### 2.4.1. Determination of PS, PDI, and ZP

The average particle diameter (z-average) and PDI of the prepared PTX-PLGA-NPs were determined by dynamic light scattering, using the photon correlation spectroscopy technique at a scattering angle of 173°. The magnitude of the surface charge of the NPs was assessed by their ZP values, which were obtained following the principles of laser Doppler velocimetry and phase analysis light scattering (M3-PALS technique). A sample of 0.2 mL of freshly prepared NPs or re-dispersed lyophilized formulation diluted with 0.8 mL of distilled water was used for the determination of both PS and ZP at room temperature using Zetasizer Nano ZS (Malvern Instruments, Malvern, UK). The values calculated were mean of three measurements (*n* = 3) and mean values ± SD were reported.

#### 2.4.2. Determination of EE%

Following preparation of PTX-PLGA-NPs, the NPs were separated from the aqueous medium by centrifugation at 15,000 rpm for 20 min. The supernatant was collected, and the separated NPs were dissolved in acetonitrile, to release the entrapped drug. The amount of entrapped drug (encapsulated PTX) in the acetonitrile solution and unentrapped drug (free PTX) in the supernatant were quantified using an Agilent 1100 series (Agilent Technologies, Wilmington, DE, USA) high performance liquid chromatography (HPLC) system, following the same procedures reported previously [36]. Briefly, the separation was achieved using a reverse phase C-18 column (Hypersil ODS, 5 µm, 4.0 × 125 mm) and a gradient mobile phase consisting of water: acetonitrile (50:50 *v*/*v*) at 0 min and (10:90 *v*/*v*) at 10 min, with a flow rate of 1.0 mL/min at 25 °C. The injection volume was 5 μL and the detection was by UV using a variable wavelength detector set at 204 nm. The HPLC method was validated for PTX assay (*R*^2^ = 0.9993), with an intra- and inter-day variation of less than 1%, and mean recovery of 99.5%. EE% of the prepared PTX-PLGA-NPs was determined according to the following equation [37]:

(1) $$EE\%=\frac{\mathrm{Encapsulated}\;\mathrm{PTX}}{\mathrm{Free}\;\mathrm{PTX}+\mathrm{Encapsulated}\;\mathrm{PTX}}\times 100$$

### 2.5. Transmission and Scanning Electron Microscopy

The morphology, size, surface structure, and topography of the optimal PTX-PLGA-NPs formulation were examined, using both transmission electron microscope (TEM) and scanning electron microscope (SEM). For TEM studies, one drop of the re-dispersed PTX-PLGA-NPs suspension was loaded on a copper-gold carbon grid and left to dry in the air. The grid was placed in the vacuum chamber of the electron microscope equipped with a LaB_6_-cathode and a Gatan Orius SC1000 camera (Gatan Inc., Pleasanton, CA, USA), at a temperature of −170 °C, using an acceleration voltage of 200 kV (JEOL-2100, Jeol Ltd., Tokyo, Japan), where photomicrographs were captured using different magnifications.

For SEM studies, one drop of the re-dispersed sample was spread on a clean slide cover and left to dry under vacuum. The dried sample was then mounted on carbon tape and sputter-coated using a gold sputter module in a high vacuum. The gold-coated samples were scanned, and photomicrographs were taken at an acceleration voltage of 3 kV using a Thermo Scientific Apreo SEM (FEI Company, Hillsboro, OR, USA).

### 2.6. Fourier Transform Infrared Spectroscopy (FT-IR)

FT-IR studies were carried out to determine any possible chemical interactions between the drug and the components of the NPs formulation, in addition to the entrapment of PTX into the NPs. FT-IR analysis of PTX, PLGA, Kol and the optimal PTX-PLGA-NPs formulation was performed using FT-IR spectrophotometer JASCO FTIR 6300 (Jasco, Easton, MD, USA). 2–3 mg of each sample was mixed with 100 mg of potassium bromide and compressed into thin discs using a hydrostatic press. The prepared samples were then scanned over a wavelength range from 4000 to 400 cm^−1^ at a resolution of 4 cm^−1^. The stretching modes and vibrational modes depict the chemical bonding and other functional groups present in the sample tested.

### 2.7. In Vitro Release Studies

The in vitro release of PTX from the optimal PTX-PLGA-NPs formulation in comparison with the free drug was determined in isopropanol: 0.9% normal saline (30:70, *v*/*v*), using a modified dialysis membrane diffusion method [38]. The release medium was chosen based on the fact that PTX is highly lipophilic, and in terms of ionization behavior is Zwitterionic, and does not have any ionizable groups with pK_a_ values in the physiological pH range. Briefly, a volume of re-dispersed PTX-PLGA-NPs suspension and free PTX solution in NMP corresponding to 200 μg PTX was placed each in a dialysis bag (molecular cut off 14,000 Da), and the dialysis bags were then soaked in 10 mL of release media in suitable glass vessels. The glass vessels were kept in an OLS Aqua Pro shaking water bath (Grant Instruments, Cambridgeshire, UK) at 37 °C ± 0.5 °C and 100 rpm. At specific time intervals (0.25, 0.5, 1, 2, 4, 6, 8, 12, 24, 48 and 72 h), 200 μL samples of release media were withdrawn and immediately replaced with fresh media. A quantitative analysis of PTX in the release media was determined using HPLC method, as described previously. Experiments were carried out in triplicate and the average cumulative percentages of PTX released were calculated using the calibration equation after correction.

The mechanism of drug release from the optimized NPs was also determined by fitting the obtained data to zero, first, Korsmeyer–Peppas, Hixson–Crowell (HC) and Higuchi diffusion equations. The linear regression equation employed for zero order kinetics was: C_t_ = C_o_ − kt; where C_o_ is the zero-time concentration of the drug, C_t_ is the concentration of the drug at time t, and k is the apparent release rate constant. First order kinetics was determined according to the equation ln C_t_ = ln C_o_ − kt. Drug release following the Higuchi model was determined using the equation Q = kt^0.5^; where Q represents the fraction of drug released in time t, and k is the Higuchi release rate constant. Drug release following the HC model was determined according to the equation ^3^√W_0_ = ^3^√W_i_ + k_HC_ t; where W_0_ is the initial amount of the drug in the system; W_i_ is the amount remaining in the system at time t; and k_HC_ is the HC release rate constant, which relates the surface area to the cubic root of volume of spherical particles. The Korsmeyer–Peppas release model was determined using the equation M_t_/M_∞_ = kt^n^; where M_t_/M_∞_ is the fraction released at time t; k is the release rate constant; and *n* is the exponent of release.

### 2.8. Short-Term Stability Study

The short-term stability of the optimal PTX-PLGA-NPs formulation (*n* = 6) was evaluated and carried out by storing the freeze-dried PTX-PLGA-NPs at room temperature or at 4 °C in refrigerator for 15 days. The vials containing PTX-PLGA-NPs lyophilizates were sealed and wrapped in aluminum foil and subdivided into two groups. One group was stored at room temperature (25 °C), and the other group was stored in refrigerator at 4 °C for 15 days. Following 15 days’ storage, samples were taken from the vials, re-suspended and subjected to PS, PDI, ZP and %EE analysis, as described above. The change in appearance (presence of aggregates), PS, PDI, ZP and %EE were recorded and compared to results obtained from freshly prepared NPs.

### 2.9. Cell Culture

Human pharyngeal squamous cell carcinoma (FaDu, ATCC^®^ HTB-43™, ATCC, Manassas, VA, USA) and human tongue squamous cell carcinoma (CAL27, ATCC^®^ CRL-2095^™^, ATCC, Manassas, VA, USA) cells were cultured in Eagle’s minimum essential medium (MEM) and Dulbecco’s modified Eagle’s medium (Sigma-Aldrich Co., St. Louis, MI, USA) respectively, supplemented with 10% heat-inactivated FBS, 100 U/mL penicillin and 100 μg/mL streptomycin under humidified air, and 5% CO_2_ at 37 °C. The cells were sub-cultivated 3 times per week at a ratio of 1:4 by trypsinization, followed by the addition of fresh growth medium.

### 2.10. Cytotoxicity and IC_50_ Studies

Cell viability studies were carried out to investigate the cytotoxic potential of the optimal PTX-PLGA-NPs formulation compared to the free drug solution. For cellular viability assays, FaDu and CAL27 cells were seeded in 96-well plates (Corning, Sigma-Aldrich Co., St. Louis, MO, USA) at a density of 5 × 10^3^ cells/well and left overnight for initial attachment. A stock solution of 10 mM of PTX in DMSO was prepared and stored at 4 °C for further use. On day 1, the cells were treated with 40 μM of PTX-PLGA-NPs, 40 μM of PTX solution in DMSO or PTX-free PLGA-NPs, for 24 h or 48 h. Fresh media, 0.4% DMSO and 1% sodium dodecyl sulphate (SDS) were used as controls. MTT (3-(4, 5-dimethyldiazol-2-yl)-2, 5-diphenyltetrazolium bromide) cell proliferation and cytotoxicity assay kit (Sigma Aldrich, St. Louis, MO, USA) was used to evaluate the anti-proliferative effect of the three treatments, after 24 h and 48 h, by adding 10 μL of 5 mg/mL MTT to each well, followed by incubation at 37 °C for 4 h. The culture media containing MTT were then removed, and 100 μL of DMSO were added to each well. The plates were shaken for 20 min, followed by measuring the optical intensity at 570 nm using a Synergy™ HTX microplate reader (BioTek, Winooski, VT, USA). Each experiment was performed in triplicate, and cell viability was expressed as the percentage of viable cells relative to the positive control using cell viability (%) = 100 A_s_/A_c_; where A_s_ is the absorbency of the samples and A_c_ is the absorbency of the control. The same procedure was repeated using different amounts of PTX-PLGA-NPs and free drug solution, to determine the concentration at which 50% of cellular growth was inhibited (inhibitory concentration (IC_50_)). The results were statistically analyzed using Prism version 8 (GraphPad Software, San Diego, CA, USA).

### 2.11. Cellular Uptake Studies

The cellular uptake of PLGA-NPs in FaDu cells was investigated by fluorescent microscopy and quantified using flow cytometry. For fluorescent microscopy, FaDu cells were seeded into 24-well plates until reaching 70% cellular confluency. CUR was used as a hydrophobic drug model instead of PTX, due to its lipophilic nature and fluorescence inherent property [39]. PLGA-NPs loaded with CUR (CUR-PLGA-NPs) were prepared using the optimized formulation with 40 μM of CUR load. CUR-PLGA-NPs were then incubated with the cultured cells for 24 h. After 24 h, the cells were washed with Phosphate Buffered Saline (PBS) (Sigma-Aldrich Co., St. Louis, MI, USA) to remove external CUR and fixed with 100 μL of ice-cold methanol and 5% acetic acid for 15 min at room temperature. The cells were then carefully washed with PBS twice, and incubated with 4′,6-diamidino-2-phenylindole (DAPI) (Sigma-Aldrich Co., St. Louis, MI, USA) nuclear stain for 10 min. Finally, the stain was removed, and the cells were imaged under a fluorescent microscope OLYMPUS-130 W U-HGLGPS (Olympus corporation, New Orleans, LA, USA). For the determination of fluorescent intensity using flow cytometry, the cells were treated with free CUR, CUR-PLGA-NPs (40 µM) or growth media as control for 24 h. After reaching 70% confluency, the cells were trypsinized and diluted with 500 μL PBS and the fluorescent intensity was determined by BD FACSAria III flow cytometer (BD Biosciences, San Jose, CA, USA). Data were acquired by BD FACSDiva software (BD Biosciences, San Jose, CA, USA) using standard fluidics, optical and electronic configuration. Data analysis was performed using FlowJo software (FlowJo LLC., Ashland, OR, USA).

### 2.12. Statistical Analysis

All in vitro experiments were performed in independent triplicates, and values are presented as mean ± SD unless otherwise noted. Statistics were carried out using Prism version 8 (GraphPad Software, San Diego, CA, USA). For comparisons between two groups, two-tailed unpaired Student’s t-test was employed. For multiple comparisons, one-way analysis of variance (ANOVA) followed by Bonferroni’s post-hoc test was utilized. A *p*-value of ≤ 0.05 was considered statistically significant.

## 3. Results and Discussion

### 3.1. Design of Experiments and Preparation of PTX-PLGA-NPs

Quality target product profiles (QTPPs) were set up considering the quality characteristics of a PTX-loaded PLGA-NPs formulation designed to be injected IT, and capable of enhancing the cellular uptake of PTX while controlling its release in cancer cells for achieving the maximal therapeutic effect. The critical process parameters (CPPs), the critical material attributes (CMAs) and CQAs were set up based on our prior knowledge in polymeric NPs formulation development and optimization in previous studies [40,41,42,43].

PTX-PLGA-NPs were successfully prepared using the nanoprecipitation method known to yield NPs with small monodisperse particles, with relative ease and low cost compared to other methodologies [44]. The ratio of the aqueous to organic phase (9:1) was used to increase the EE% and further reduce PS [45]. Kol, an FDA approved pharmaceutical ingredient, was used as stabilizer, due to its undeniable good surfactant and solubilizing properties, that made its use favorable in many parenteral formulations [19]. NMP was selected as an organic solvent, as it is biodegradable, safe to use and possess a high solvation power for PTX. A significant decrease in PS and PDI was achieved in this study using NMP compared to other solvents such as acetone [35], thus highlighting the importance of organic solvent selection in NPs optimization. The lyophilization of the optimal PTX-PLGA-NPs formulation resulted in the formation of dry powders that were easily re-dispersible.

The process parameters and the lyophilization technique used in the preparation of PTX-PLGA-NPs were selected based on our previous knowledge from previous studies, as stated before. The response data of the 22 experimental runs using the previously described experimental design are presented in Table 2.

#### 3.1.1. Determination of PS, PDI and ZP

**Figure 1:**
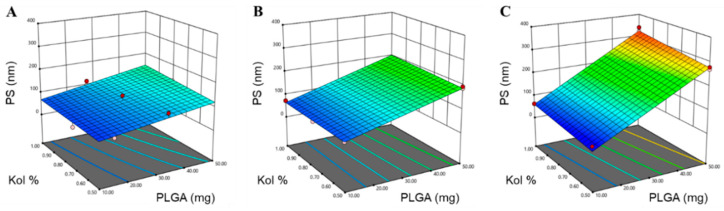
Response surface plots for the effect of the amount of PLGA (mg) and Kol % (*w*/*v*) on PS using (**A**) PLGA-7K, (**B**) PLGA-24K and (**C**) PLGA-38K.

**Figure 2:**
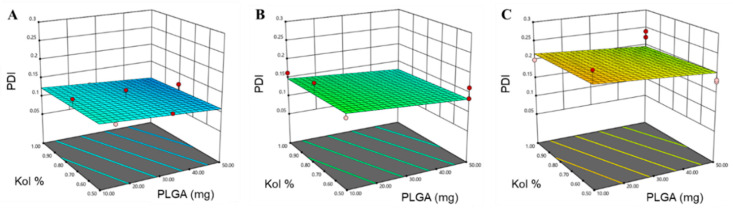
Response surface plots for the effect of the amount of PLGA (mg) and Kol % (*w*/*v*) on PDI, using (**A**) PLGA-7K, (**B**) PLGA-24K and (**C**) PLGA-38K.

**Figure 3:**
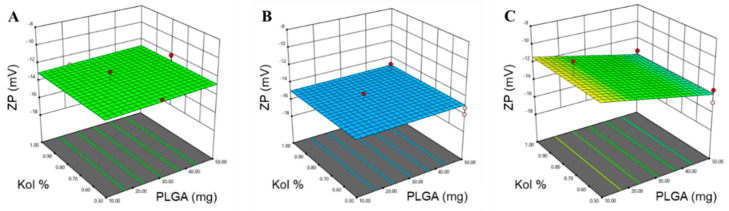
Response surface plots for the effect of the amount of PLGA (mg) and Kol % (*w*/*v*) on ZP using (**A**) PLGA-7K, (**B**) PLGA-24K and (**C**) PLGA-38K.

To deconvolute the individual effects of the three independent factors and their interactions on the selected responses, the data were statistically analyzed and multiple linear regression analysis and ANOVA were employed to model the data and develop a mathematical expression in the form of a second order polynomial equation, as described below:

(2) $$Y=\beta_{o}+\beta_{1}X_{1}+\beta_{2}X_{2}+\beta_{3}X_{3}+\beta_{11}X_{1}^{2}+\beta_{22}X_{2}^{2}+\beta_{33}X_{3}^{2}+\beta_{12}X_{1}X_{2}+\beta_{13}X_{1}X_{3}+\beta_{23}X_{2}X_{3}$$

where *Y* is the response, *β_ο_*, *β*_1_, *β*_2_, *β*_3_, …, *β*_23_ are the regression coefficients and *X*_1_, *X*_2_, *X*_3_, are the studied factors at the specified levels. The equation in terms of coded factors can be used to make predictions about the response for the given levels of each factor and can be useful for identifying the relative impact of the factors by comparing the factor regression coefficients. Three and higher-order interactions were neglected. The predicted *R*^2^ values were in a reasonable agreement with the adjusted *R*^2^ for all responses, indicating that the model has predicted all responses values well (Table 3). The adequate precision, which measures the signal-to-noise ratio to ensure that the model can be used to navigate the design space, was greater than 4 (the desirable value) for all responses. The graphical analysis of the effects of the formulation variables on PS, PDI and ZP is shown in Figure 1, Figure 2 and Figure 3.

The particle size is a very critical attribute, which affects stability, encapsulation efficiency, drug release profile, pharmacokinetics, biodistribution and cellular uptake [40,41,46,47]. The use of different polymers in the formulation of NPs, as well as the use of different cell lines to simulate the in vivo uptake/absorption, contribute largely to the inconsistency in the reported cut-off sizes able to cross biological/cellular membranes [48,49]. Nevertheless, it is acknowledged that the smaller the PS is, the greater the extent of cellular uptake will be.

The average PS, presented as z-average diameter, of the 22 formulations ranged from 42 to 339 nm with PDI values, ranging from 0.075 to 0.256, as demonstrated in Table 2. The wide range of PS and PDI observed indicates that the studied formulation variables are critical and have a significant effect on the analyzed response variables. As shown in Figure 1 and Table 3, the three tested formulation variables had a significant positive impact on the mean PS (*p* < 0.0001).

Moreover, the ANOVA test showed a significant two-factor interaction for the effect of the amount of PLGA and Mw of PLGA (*X*_1_*X*_3_) on the PS (*p* < 0.0001), indicating the rather complex statistical model for PS. The PS equation obtained from the analysis was:

PS = 133.71 + 77.37 *X*_1_ + 10.17 *X*_2_ − 34.76 *X*_3_ − 3.31 *X*_3_ + 6.31 *X*_1_*X*_2_ − 40.08 *X*_1_*X*_3_ − 0.9859 *X*_2_*X*_3_ (3)

According to the statistical analysis, an increase in the amount of PLGA resulted in a significant increase in the average PS of the NPs (*p* = 0.0001), as depicted in Figure 1. This might be attributed to the increase in the amount-to-volume ratio and possible aggregation of the NPs associated with an increase in the compression of the electric double layer, thus decreasing the repulsive strength of the charged particles and their inherent stability [50]. Furthermore, upon the addition of more PLGA, the viscosity of the organic solvent increases, providing resistance to the diffusion of the polymer solvent phase into the aqueous phase, henceforth, producing larger sized NPs [50]. The higher viscosity of the organic phase is also expected to increase polymer-polymer and polymer-solvent interactions, which might result in the formation of larger particles [51,52]. The results also suggest a moderate inconclusive evidence (*p* = 0.024) that the size of PTX-PLGA-NPs increases with the increase in the concentration of Kol. Surfactants tend to accumulate and adsorb onto the surface of the NPs, due to their amphiphilic nature which might result in an increase in the size of the particles. This phenomenon is known as oriented physical adsorption, and it prevents the interaction between particles, hence reducing their aggregation and improving their stability [53]. In addition, increasing the concentration of Kol might enhance the solubility of PLGA in the solvent-system, thus reducing the rate of PLGA precipitation, resulting in the formation of larger particles. Finally, the effect of the Mw of PLGA on PS was rather more complicated. At a low amount of PLGA, the PS was not affected by the Mw of PLGA; on the contrary, the PS was significantly increased when higher amounts of PLGA were combined with the use of higher Mw PLGA, especially 24K and 38K, reaching a maximum size when high amount of PLGA-38K was used in the fabrication of the NPs. The two-factor interaction between the amount of PLGA and Mw of PLGA might be attributed to the increasing polymeric chain length of higher Mw PLGA compared to lower Mw PLGA, resulting in an increase in the hydrophobic interactions between the longer chains, and the formation of bulkier structures, with these interactions becoming more prominent as the amount of the polymer used is increased [45].

A more uniform size distribution of particles was reported to provide a higher physical stability [54]. The average PDI values of the prepared NPs formulations ranged from 0.075 to 0.256, as reported in Table 2. The polynomial equation produced for PDI was:

PDI = 0.1543 − 0.0085 *X*_1_ − 0.0020 *X*_2_ − 0.0381 *X*_3_ − 0.0135 *X*_3_ (4)

As presented in Table 3 and graphically illustrated in Figure 2, the Mw of PLGA was the only factor producing a significant effect on the PDI response (*p*-value < 0.0001), where using high Mw PLGA was associated with higher PDI. This could be due to the increase in the polymer chain length variability known to be associated with higher Mw polymers relative to lower Mw polymers, thus increasing the size distribution of the NPs [45]. Additionally, it is well documented in the literature that smaller particles are associated with lower PDI, while larger particles tend to have higher PDI, taking into consideration the composition of the nanocarrier and the nature of the solvent used in the fabrication of the NPs [55,56,57].

The ZP measurements revealed that all NPs formulations carried a negative charge in agreement with the polyanionic nature of PLGA, due to negatively charged carboxylic functional groups (Table 2). The mean ZP values of the prepared PTX-PLGA-NPs formulations were in the range of −10 to −16 mV, which could indicate a lower risk of aggregation and a high level of physical stability as a result of repulsive forces between the NPs [31]. Here again, only the Mw of PLGA was associated with a significant change in the ZP of the NPs (*p* = 0.0039); however, a two-factor interaction was still evident between the effect of the amount of PLGA and the Mw of PLGA (*X*_1_*X*_3_) on the ZP (*p* = 0.0298).

The polynomial equation produced for the ZP was:

ZP = −13.43 − 0.4509 *X*_1_ + 0.4741 *X*_3_ − 1.52 *X*_3_ + 0.6481 *X*_1_*X*_3_ (5)

As presented in Table 3 and graphically illustrated in Figure 3, the use of PLGA-24K in the fabrication of the NPs was associated with the highest ZP compared to PLGA-7K and PLGA-38K, which could be due to the proper orientation of the carboxylic groups in this specific chain length on the surface of the NPs compared to other chain lengths.

Surprisingly, the ZP of NPs fabricated with PLGA-38K gradually increased (became more negative) with the use of higher amount of PLGA. This might be due to the large increase in PS, as described before, leading to an increase in the total number of free carboxylic groups on the surface of the NPs hence an increased ZP. Moreover, the reduced ZP associated with the use of small amount of PLGA-K38 can be attributed to the fact that longer polymer chains mean less carboxylic acid groups per unit mass, therefore less carboxylate groups or negative charges. Another explanation may arise from the insignificant effect of the concentration of the non-ionic stabilizer Kol on the ZP response, indicating that the surfactant layer is not densely packed in the concentration range studied, thus not resulting in any significant coverage of the negative charge of PLGA. Therefore, at any given concentration, assuming that the surface is not fully packed with the surfactant molecules, more surfactant molecules will be adsorbed per unit area in the case of smaller particles as compared to larger particles. Higher adsorption density should impart greater shielding effect of the negative charges, and hence smaller negative ZP values will be observed in the case of smaller NPs. The assumption is supported by the observation that the simultaneous increase in the amount and Mw of PLGA significantly increases the PS.

#### 3.1.2. Determination of EE%

The amount of drug encapsulated into a nanoparticulate system is crucial, as it will determine the amount of the NPs formulation, equivalent to a certain dose, to be used in therapy. An extremely high EE% reduces the amount of excipients, used in the fabrication of NPs, to be administered especially high amounts of surfactants known to be associated with in vivo toxicity, in addition to reducing the size of the dosage form to be administered and cost of manufacturing.

The percent PTX entrapped in the different NPs formulations was highly variable and ranged from 57% to 95% (Table 2). Statistical analysis showed that only the Mw of PLGA had a significant impact on the drug EE% (Table 3). The polynomial predictive equation produced for the EE% was:

EE% = 72.62 − 5.24 *X*_3_ − 3.57 *X*_3_ (6)

**Figure 4:**
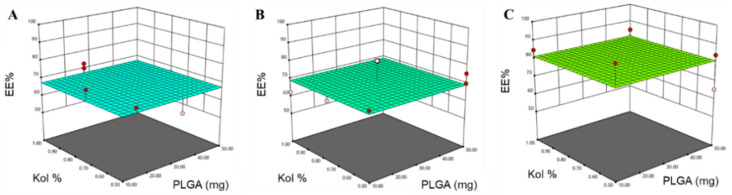
Response surface plots for the effect of the amount of PLGA (mg) and Kol % (*w*/*v*) on EE% using (**A**) PLGA-7K, (**B**) PLGA-24K and (**C**) PLGA-38K.

As depicted in Figure 4, the use of high Mw PLGA resulted in a significant increase in the percentage drug entrapped within the NPs (*p* = 0.0039).

The Mw is a very important parameter that modifies the physico-chemical properties of the polymer and the nanoparticle dispersion. Moreover, the Mw strongly influences the capacity of drug encapsulation and/or adsorption on the nanoparticle surface, due to differences in the magnitude of the electrostatic and hydrophobic interactions. These interactions are governed by the length of the polymeric chains. The level of significance of the Mw of PLGA on EE% suggests a direct interaction between high Mw PLGA and PTX, which affect the EE% of PTX. This could be attributed to the higher lipophilicity associated with high Mw PLGA due to higher polymeric chains lengths, and considering the hydrophobicity of PTX, it is reasonable to say that PTX might undergo hydrophobic interaction with high Mw PLGA polymer chains compared to lower Mw PLGA resulting in higher EE% [45]. As mentioned earlier, NPs prepared using PLGA-38K produced extremely small PS when combined with the use of low amount of PLGA, suggesting the ability of PLGA-38K to increase the encapsulation of the drug to a large extent without compromising the PS.

### 3.2. Design Space and Model Verification

**Figure 5:**
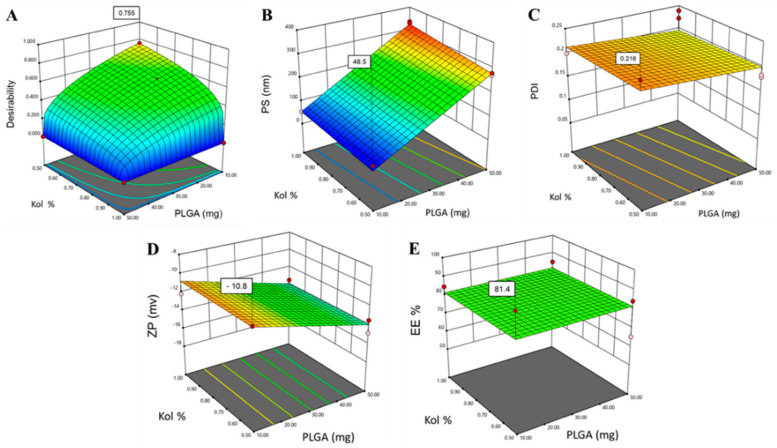
Optimization of PTX-PLGA-NPs showing response surface plots for the effect of using 10 mg PLGA-K38 and 0.5% Kol on (**A**) the desirability value, (**B**) PS (nm), (**C**) PDI, (**D**) ZP (mV) and (**E**) EE%.

The response surface analysis of the D-optimal surface design was used to predict the optimum levels of the studied factors for the preparation of PTX-PLGA-NPs (Figure 5). The target was to minimize PS (<60 nm) and PDI and maximize EE% and ZP. Simultaneous optimization of all responses was performed. The desirability value was used to select the optimal formulation where the PS and EE% were given the highest priority followed by PDI and ZP. The highest desirability value (0.755), as shown in Figure 5A, was depicted in the formulation prepared using 10 mg PLGA-K38 and 0.5% *w*/*v* Kol (F16 in Table 2).

The optimal selected formulation produced an observed z-average PS of 53.1 ± 2.8 nm, PDI of 0.221 ± 0.017, ZP of −10.1 ± 1.2 mV and EE% of 92.2 ± 3.9% (Table 4). Based on these results, it can be concluded that the optimal PTX-PLGA-NPs formulation, guided by QbD, provides a promising formulation for the easy production of ultra-high drug-loaded PLGA-NPs with ultra-small size; therefore, it was selected for further investigations. The optimal formulation was also used to prepare PTX-free PLGA-NPs to be used in some of the investigations. PTX-free PLGA-NPs had a mean PS, PDI and ZP of 64.4 ± 4.5 nm, 0.201 ± 0.016 and −13.8 ± 3.33 mV, respectively.

### 3.3. TEM and SEM Analysis

**Figure 6:**
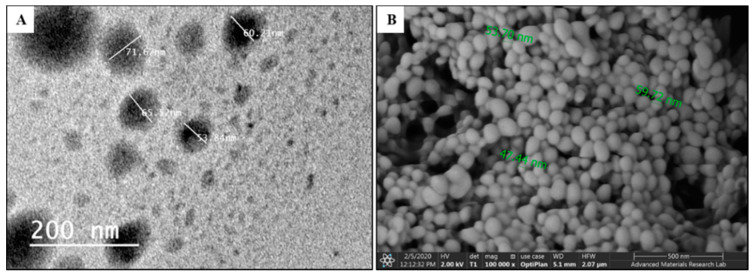
Morphology, surface structure and size of the optimal PTX-PLGA-NPs formulation observed using (**A**) TEM micrograph; (**B**) SEM micrograph.

The morphology, surface structure, and topography of the optimal PTX-PLGA-NPs formulation were investigated through TEM and SEM imaging, as illustrated in Figure 6.

TEM and SEM micrographs of the optimal PTX-PLGA-NPs formulation showed spherical, smooth and uniformly shaped particles. The size was within the range of 40 nm to 70 nm. This aligns with the previous PS measurements obtained through the Zetasizer. The PS and PDI of the nanocarrier system were reported to be the main physicochemical attributes that influence endocytosis-dependent cellular uptake [55]. Previous studies also showed the superior penetration and retention behavior of NPs less than 50 nm in tumors, in addition to reduced mononuclear phagocyte-system (MPS)-mediated clearance following systemic administration [58]. Therefore, TEM and SEM results might confirm the suitability of the optimal formulation to achieve enhanced cellular uptake, and to accumulate efficiently in even poorly permeable tumors.

### 3.4. FT-IR Analysis

**Figure 7:**
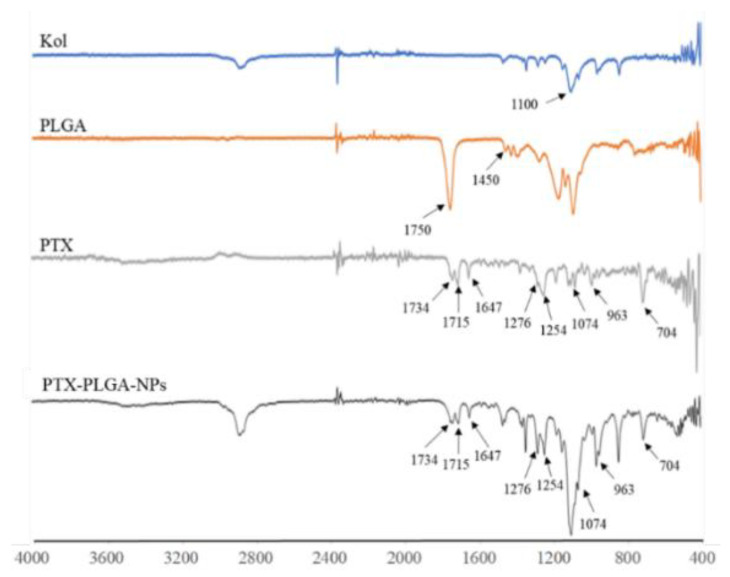
The FT-IR spectra of PTX, PLGA, Kol and optimized PTX-PLGA-NPs.

The potential interaction between the drug and the materials used in the fabrication of the NPs was assessed using FT-IR. The FT-IR spectra of PTX, PLGA, Kol and PTX-PLGA-NPs are depicted in Figure 7.

Major IR peaks assigned to PTX were observed at 1734 cm^−1^ and 1715 cm^−1^ (C=O); 1647 cm^−1^ (amide bond); 1074 cm^−1^ (C–O), 963 cm^−1^ and 704 cm^−1^ (aromatic C=C). In addition, bands at 1254 cm^−1^ and 1276 cm^−1^ were attributed to ester bond stretching and C-N stretching vibrations, respectively. PLGA showed characteristic peaks at 1750 cm^−1^ (C=O) and 1455 cm^−1^ (CH_2_−), while Kol showed a characteristic absorption peak at 1100 cm^−1^ (C–H). Spectral analysis of PTX-PLGA-NPs showed no spectral shift for PTX, but was merely a superposition of PTX characteristic peaks, indicating the absence of any chemical interaction between PTX and the other components used in the fabrication of the NPs.

### 3.5. In Vitro Release Studies

**Figure 8:**
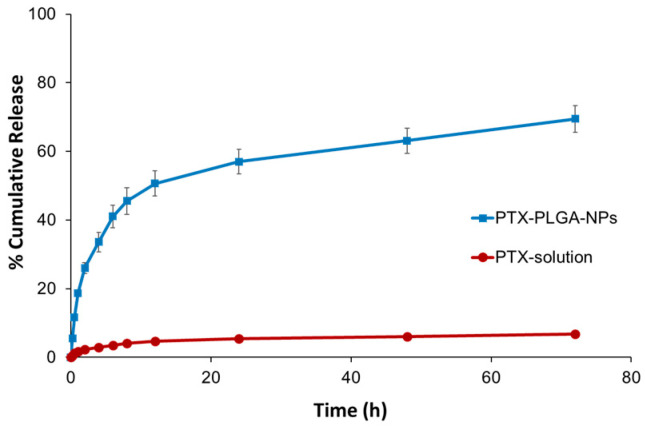
In vitro release profiles of PTX from the optimized PTX-PLGA-NPs formulation and its solution in NMP in isopropanol: 0.9% normal saline (30:70, *v*/*v*) at 37 °C. Data points are mean ± SD (*n* = 3).

Drug release characteristics from polymeric NPs are known to depend on the dissolution rate of the encapsulated drug, followed by its diffusion out of the NPs in addition to other factors related to NPs swelling and erosion/degradation in the release medium. The in vitro release profile of PTX from the optimized PTX-PLGA-NPs in comparison to PTX solution in NMP is shown in Figure 8.

PTX release from PTX-PLGA-NPs showed a characteristic biphasic release pattern. The first phase was characterized by a relatively rapid release of the drug from the NPs, where almost 50% of the drug was released in the first 12 h. The second phase, on the other hand, was characterized by a slower drug release at a constant rate, that was sustained over the next 60 h. After 72 h, 70% of the drug was released from the NPs through the dialysis membrane, compared to only 6.8% from the free drug solution in NMP. The relatively rapid drug release observed over the first 12 h might be due to the extremely small PS, presenting a huge surface area of contact with the release medium, thus allowing drug present close to the surface of the particles to be rapidly released. On the other hand, more deeply embedded drug is released later at a slower rate, thus providing a sustained release profile for the drug, where the release behavior is also governed by other factors, such as the redistribution of PTX inside the NP matrix, in addition to changes in the surface porosity and internal morphology of the NPs, all of which have been reported to be important parameters that can alter the release profile of the encapsulated drug [35,59]. The kinetics of drug release over the first 24 h from the optimal NPs formulation were also subjected to statistical regression analysis, according to five different models, and the release kinetics were found to best fit the Korsmeyer–Peppas model equation (Table 5), suggesting that the drug release from the polymeric NPs matrices is occurring via multiple mechanisms, amongst which is the diffusion and polymeric chain relaxation [44,60].

### 3.6. Short-Term Stability Study

Nanosystems are highly energetic systems and thermodynamically unstable. These systems tend to aggregate to lower their surface free energy. Although the use of stabilizers in addition to freeze-drying is known to improve the physical stability of nanosuspensions, the stresses encountered in the freeze-drying process might result in several changes in the CQAs of the freeze-dried product. There was no statistical difference in the PS, PDI, ZP and EE% of the lyophilized PTX-PLGA-NPs when stored in a refrigerator at 4 °C for 15 days, when compared to the freshly prepared NPs (Table 6), indicating the short-term stability of the optimal PTX-PLGA-NPs formulation and its resistance to the freeze-drying stresses. On the other hand, a significant increase in PS and PDI along with a decrease in ZP was observed after 15 days storage at room temperature, with no change in EE%. In this work, all investigations were carried out using either freshly prepared NPs or lyophilizates stored for no more than 15 days at 4 °C. 

### 3.7. In Vitro Cytotoxicity and Cellular Uptake Studies

**Figure 9:**
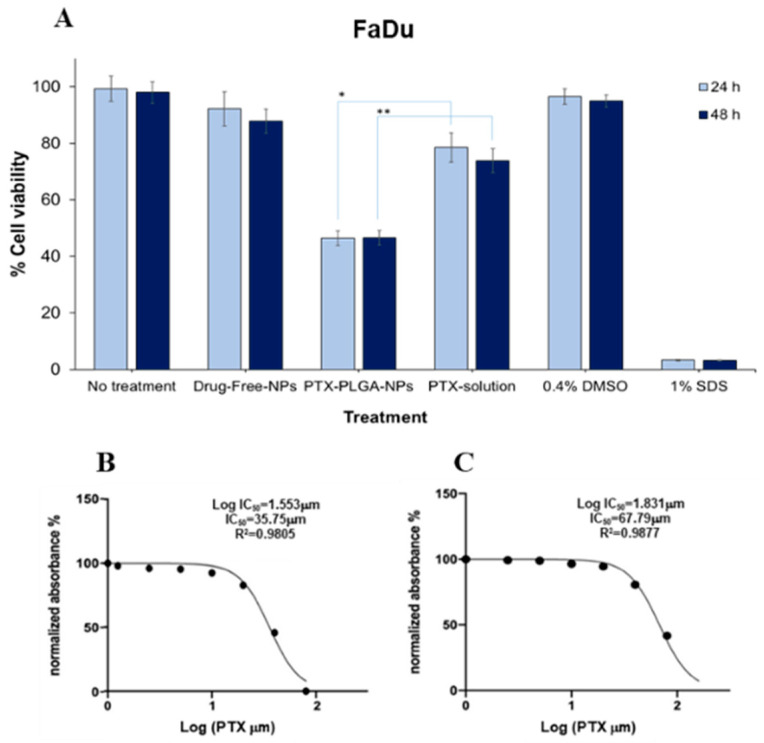

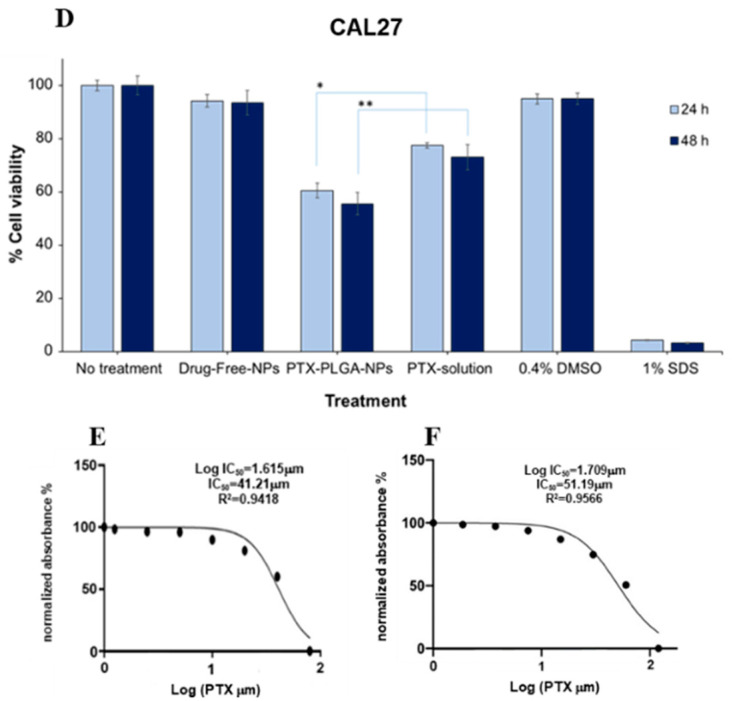
Determination of % cell viability (**A**) In vitro cytotoxicity results using FaDu cells treated for 24 h and 48 h; (**B**) Determination of IC_50_ for optimized PTX-PLGA-NPs and (**C**) free PTX solution using FaDu cells; (**D**) In vitro cytotoxicity results using CAL27 cells treated for 24 h and 48 h; (**E**) Determination of IC_50_ for optimized PTX-PLGA-NPs and (**F**) free PTX solution using CAL27 cells. * *p* < 0.05, ** *p* < 0.01.

A common drawback in traditional delivery systems is the inability to encompass the wide scope of side effects due to systemic and non-specific administration, especially with hard-to-reach targets. The present study aimed to optimize the features of PTX-PLGA-NPs as a drug delivery system to improve PTX efficacy as a model drug for the treatment of HNSCC, especially when injected intratumorally. This can be achieved by enhancing the cellular uptake of PTX through the proposed PTX-PLGA-NPs formulation, as well as offering physical protection of the encapsulated drug against enzymatic activity in the tumor cells, thus increasing the residence time of the drug at the tumor site upon IT injection. FaDu and CAL27 cell lines are commonly used in vitro cellular models for the study of chemotherapeutic agents in the treatment of HNSCC [59,61]. Previous work on FaDu cell lines using PTX have demonstrated the moderate efficacy of this drug on the cell line [62]. In order to determine whether FaDu cells were able to effectively uptake PTX-PLGA-NPs, and whether the NPs were able to deliver PTX into the FaDu cells, cellular cytotoxicity and uptake studies were performed. The cytotoxic potential of the optimal PTX-PLGA-NPs relative to PTX-solution and PTX-free PLGA-NPs was assessed by performing MTT assays. Remarkable differences in cell survival were observed from the three treatments following 24 h and 48 h of treatment, as depicted in Figure 9A. Similar results were obtained upon treatment of CAL27 cells with PTX-PLGA-NPs (Figure 9D). Treating the cells with PTX-free PLGA-NPs did not result in any significant decrease in % cell viability relative to the positive control, indicating the biocompatibility and non-toxicity of the materials used in the fabrication of the NPs. Treating FaDu cells with PTX-PLGA-NPs resulted in statistically significantly higher cytotoxic effects compared to the free drug solution (*p* = 0.0022), reducing the cell viability by more than 50% in 24 h. The average % cell viability at 24 h obtained from PTX-PLGA-NPs treatment was 46 ± 2.6% compared to an average of 79 ± 5.2% from treating the cells with the free drug solution. The significant decrease in cell survival by almost 50% in 24 h using PTX-PLGA-NPs compared to only 20% using the free drug solution indicates that the cells respond and interact differently to the chemotherapeutic agent when delivered in the form of NPs compared to free drug solution. Similarly, treatment of CAL27 cells with PTX-PLGA-NPs resulted in a significant decrease in cell viability compared to treatment with PTX- solution (*p* = 0.005), confirming the enhanced cytotoxic effect of the optimized NPs.

Results also showed that the cytotoxic effect of PTX-PLGA-NPs was maintained after the 48-h treatment in both cell lines, which could be due to the sustained release phase of the drug from the NPs, where maintenance of an effective drug concentration for a prolonged period of time is essential for many therapeutic regimens. This might also indicate that the cytotoxic effect obtained from PTX-PLGA-NPs is concentration dependent and not time dependent, since the cytotoxic effect of the free drug was slightly increased after the 48-h treatment, even though these results were not statistically different from the 24-h results.

The chemotherapeutic efficacy of the drug was further investigated by measuring the IC_50_ of PTX-PLGA-NPs compared to the free drug solution in FaDu cells, as depicted in Figure 9B,C. Results showed that the optimized PTX-PLGA-NPs formulation had a significantly lower IC_50_ (35.75 μM) compared to the free drug solution (67.79 μM). Similarly, the IC_50_ of the optimized PTX-PLGA-NPs (41.21 μM) was significantly lower than that of the PTX-solution (51.19 μM) in CAL27 cells, confirming the ability of the optimal formulation to increase the therapeutic efficiency of PTX. This effect might be due to the enhanced cellular uptake of PTX when efficiently encapsulated in NPs with optimized PS and PDI.

The cellular uptake or internalization of actives is one of the most important physico-chemical parameters to be considered prior to in vivo application. The cellular uptake of the optimal NPs formulation was investigated by fluorescent microscopy and flow cytometry, using CUR as a model drug due to its lipophilic nature and fluorescence inherent property; thus serving as a hydrophobic drug model instead of PTX.

**Figure 10:**
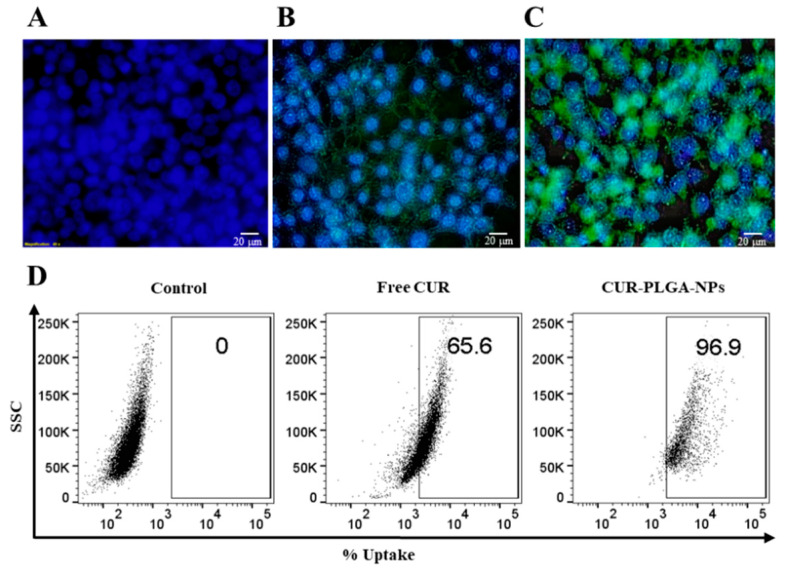
Cellular uptake studies in FaDu cells showing qualitative analysis of cellular localization (**A**) control (no treatment); (**B**) free CUR; (**C**) optimized CUR-PLGA-NPs (magnification 40×); and (**D**) quantitative analysis of cellular uptake by flow cytometry.

Results revealed a significant increase in the intracellular accumulation of CUR-PLGA-NPs, where the cellular uptake in FaDu cells was about 33% higher from the optimal NPs formulation compared to the free CUR solution, as depicted in Figure 10. These results indicate that the optimal PTX-PLGA-NPs formulation was able to achieve marked enhancement in the cellular uptake and eventually higher efficacy of PTX. These results are also in accordance with previous studies, in which it was reported that particles < 70 nm tend to accumulate more in tumor cells and be retained intracellularly [63].

Altogether, these results indicate that the proposed PTX-PLGA-NPs formulation, optimized using optimal CQAs including PS, PDI, ZP and EE%, significantly increased the cellular uptake of PTX in head and neck cancer cell lines. The optimized formulation is particularly attractive for the treatment of HNSCC by IT injection, because of its potential of delivering an adequate drug dose directly to the target site, in addition to sustaining the drug concentration in the tumor cells at effective levels without undesirable systemic side effects. The favorable attributes may also include biocompatibility, timely drug release, prolonged residence time, and possibly deeper tumor penetration, with effective endosomal escape leading to the delivery of maximal amount of encapsulated drug to the tumor cells, and eventually higher therapeutic efficacy.

Future studies will focus on testing PTX-PLGA-NPs delivery to head and neck cancer animal model following IT injection. The pharmacodynamic and pharmacokinetic responses obtained following the IT injection will also be compared to those obtained following an intravenous administration of PTX in the form of PLGA-NPs or simple solution.

## 4. Conclusions

In this study, ultra-high drug loaded PTX-PLGA-NPs with ultra-small size were successfully prepared using a modified nanoprecipitation technique, and the use of NMP as a solvent was beneficial in decreasing the PS relative to more common organic solvents reported in many studies. The QbD approach was very effective in analyzing the main as well as the interaction effects of the studied factors on the selected responses with a small number of experimental runs. The amount and Mw of PLGA as well as the concentration of the stabilizer, with an evident two-factor interaction, were identified as critical parameters affecting the PS of PTX-PLGA-NPs, while PDI, ZP and EE% were mainly affected by the Mw of PLGA. QbD experiments also demonstrated that the use of high Mw PLGA increased the EE% significantly without compromising the PS. From these results, a design space was defined and generated to get PTX-PLGA-NPs with desired physico-chemical characteristics. In vitro release studies from optimized formulation showed the ability of the NPs to improve the release of PTX in a controlled manner. A substantial increase in the efficacy of PTX in terms of cellular cytotoxicity and uptake relative to the free drug in HNSCC was achieved. This study highlights the great potential of the optimal NP formulation to serve as a delivery platform for producing a variety of colloidal systems with ultrahigh encapsulation efficiency and ultra-small size, for the delivery of a vast majority of chemotherapeutic agents to tumor cells.

## Figures and Tables

**Table 1:** Independent variables (factors) and dependent variables (responses) for the optimization of ultra-high paclitaxel-loaded poly(lactide-*co*-glycolide) nanoparticles (PTX-PLGA-NPs).

Numerical Factors		Applied Levels			
		Low (−1)		High (+1)	
*X*_1_	Amount of PLGA (mg)	10		50	
*X*_2_	Kol concentration (g/100 mL)	0.5		1	
**Categorical Factor**		**Applied Levels**			
*X*_3_	Mw of PLGA (kD)	7–17	24–38		38–54
**Responses**		**Optimization Goal**			
*Y*_1_	Particle size (PS) (nm)	<60 nm			
*Y*_2_	Polydispersity index (PDI)	Minimize			
*Y*_3_	Zeta potential (ZP) (mV)	Maximize			
*Y*_4_	Encapsulation efficiency (EE) (%)	Maximize			

**Table 2:** Experimental design and measured responses for the optimization of PTX-PLGA-NPs.

Formulation	*X*_1_	*X*_2_	*X*_3_	*Y*_1_: PS (nm)	*Y*_2_: PDI	*Y*_3_: ZP (mV)	*Y*_4_: EE (%)
F1	50	1	38	329.4 ± 2.9	0.239 ± 0.048	−13.5 ± 2.1	84.9 ± 3.6
F2	50	1	38	335.8 ± 3.1	0.222 ± 0.015	−14.4 ± 2.6	80.4 ± 4.7
F3	29.2	0.8	7	118.7 ± 1.7	0.139 ± 0.014	−11.8 ± 2.2	65.4 ± 3.9
F4	10	1	24	72.5 ± 0.4	0.163 ± 0.011	−13.5 ± 1.8	62.3 ± 5.1
F5	10	0.8	24	61.8 ± 0.7	0.181 ± 0.016	−16.0 ± 3.9	66.6 ± 2.8
F6	15	0.5	7	58.9 ± 0.4	0.122 ± 0.011	−12.5 ± 2.1	70.0 ± 4.1
F7	50	0.5	38	278.1 ± 2.1	0.179 ± 0.015	−13.5 ± 1.4	84.1 ± 3.8
F8	20	0.8	38	79.4 ± 0.9	0.171 ± 0.013	−9.9 ± 1.6	75.8 ± 2.2
F9	50	0.8	7	130.0 ± 1.4	0.092 ± 0.008	−12.1 ± 1.7	66.8 ± 3.5
F10	46.3	0.9	24	184.2 ± 1.9	0.106 ± 0.009	−13.8 ± 2.1	57.3 ± 2.6
F11	50	0.5	24	193.7 ± 1.5	0.163 ± 0.012	−16.0 ± 2.3	75.3 ± 5.5
F12	10	1	38	62.7 ± 0.7	0.199 ± 0.014	−12.2 ± 1.4	84.7 ± 5.3
F13	26.8	1	7	97.3 ± 1.0	0.111 ± 0.011	−13.0 ± 2.9	72.8 ± 3.7
F14	33.2	0.5	7	113.2 ± 1.1	0.121 ± 0.017	−12.7 ± 1.6	59.4 ± 2.7
F15	50	0.8	7	115.3 ± 1.1	0.121 ± 0.012	−13.9 ± 2.9	61.1 ± 4.3
F16	10	0.5	38	51.1 ± 0.8	0.256 ± 0.019	−10.7 ± 1.9	95.0 ± 4.6
F17	10	0.5	24	66.2 ± 1.3	0.144 ± 0.016	−15.9 ± 2.3	70.9 ± 3.4
F18	26.8	1	7	116.4 ± 1.9	0.075 ± 0.015	−13.6 ± 2.1	70.1 ± 2.9
F19	30	0.8	24	121.9 ± 2.1	0.098 ± 0.008	−14.1 ± 1.9	81.2 ± 4.4
F20	50	0.5	24	186.8 ± 1.6	0.135 ± 0.018	−15.3 ± 2.4	69.8 ± 3.9
F21	10	0.7	7	42.3 ± 0.6	0.148 ± 0.016	−14.1 ± 1.3	73.5 ± 4.1
F22	50	0.5	38	269.4 ± 2.8	0.183 ± 0.020	−14.9 ± 1.1	65.3 ± 2.8

Data are mean values ± SD (*n* = 3).

**Table 3:** Output results of the experimental design.

Response	*R* ^2^	Adjusted *R*^2^	Predicted *R*^2^	Adequate Precision	Significant Terms
PS (nm)	0.9801	0.9651	0.9151	25.859	*X*_1_ (*p* < 0.0001) *X*_2_ (*p* = 0.024) *X*_3_ (*p* < 0.0001) *X*_1_*X*_3_ (*p* < 0.0001)
PDI	0.7036	0.6339	0.5160	8.222	*X*_3_ (*p* < 0.0001)
ZP (mV)	0.6623	0.5568	0.3101	7.4293	*X*_3_ (*p* = 0.0016) *X*_1_*X*_3_ (*p* = 0.0298)
EE (%)	0.4416	0.3828	0.2453	5.0564	*X*_3_ (*p* = 0.0039)

**Table 4:** Optimized parameters along with predicted and observed values of responses.

Variables	Values	Response	Predicted Values	Observed Values
*X*_1_	10 mg	*Y*_1_ (PS)	48.5 nm	53.1 nm
*X*_2_	0.5% (*w*/*v*)	*Y*_2_ (PDI)	0.22	0.22
*X*_3_	38–54 kD	*Y*_3_ (ZP)	−10.8 mV	−10.1 mV
		*Y*_4_ (EE%)	81.4%	92.2%

**Table 5:** Mechanism of PTX release from the optimal PTX-PLGA-NPs formulation.

Release Kinetic Model	Equation	k	Unit	*n*	*R* ^2^
Zero Order	C_t_ = C_o_ − kt	2.2156	%/h	-	0.713
First Order	ln C_t_ = ln C_o_ − kt	0.0343	h^−1^	-	0.801
Korsmeyer–Peppas	M_t_/M_∞_ = kt^n^	15.495	h^−*n*^	0.4936	0.941
Hixson–Crowell	^3^√W_0_ = ^3^√W_i_ + k_HC_ t	0.0457	(%)^1/3^/h	-	0.772
Higuchi	Q = kt^0.5^	12.57	(%)/h^0.5^	-	0.933

**Table 6:** Mean ± (SD) particle size (PS), polydispersity index (PDI), zeta potential (ZP) and entrapment efficiency (EE%) of the optimal PTX-PLGA-NPs formulation after 15 days storage at room temperature and 4 °C.

Formulation	Storage Conditions	PS (nm)	PDI	ZP (mV)	EE (%)
PTX-PLGA-NPs	Fresh	51.7 ± 1.7	0.207 ± 0.024	−11.6 ± 1.6	93.9 ± 3.7
	25 °C	322.2 * ± 23.8	0.608 * ± 0.118	−8.1 * ± 2.7	95.1 ± 4.7
	4 °C	54.3 ± 3.4	0.249 ± 0.040	−11.2 ± 1.6	91.2 ± 3.3

Data are mean values (*n* = 6) ± SD; * *p* < 0.05.
